# Selecting PCR for the Diagnosis of Intestinal Parasitosis: Choice of Targets, Evaluation of In-House Assays, and Comparison with Commercial Kits

**DOI:** 10.1155/2017/6205257

**Published:** 2017-08-30

**Authors:** G. N. Hartmeyer, S. V. Hoegh, M. N. Skov, R. B. Dessau, M. Kemp

**Affiliations:** ^1^Research Unit of Clinical Microbiology, Institute of Clinical Research Faculty of Health Science, University of Southern Denmark, Odense, Denmark; ^2^Department of Clinical Microbiology, Odense University Hospital, Odense, Denmark; ^3^Department of Clinical Microbiology, Slagelse Hospital, Slagelse, Denmark

## Abstract

Microscopy of stool samples is a labour-intensive and inaccurate technique for detection of intestinal parasites causing diarrhoea and replacement by PCR is attractive. Almost all cases of diarrhoea induced by parasites over a nine-year period in our laboratory were due to* Giardia lamblia*,* Cryptosporidium *species, or* Entamoeba histolytica* detected by microscopy. We evaluated and selected in-house singleplex real-time PCR (RT-PCR) assays for these pathogens in 99 stool samples from patients suspected of having intestinal parasitosis tested by microscopy. The strategy included a genus-specific PCR assay for* C. parvum* and* C. hominis*, with subsequent identification by a PCR that distinguishes between the two species.* G. lamblia* was detected in five and* C. parvum *in one out of 68 microscopy-negative samples. The performance of the in-house RT-PCR assays was compared to three commercially available multiplex test (MT-PCR) kit systems in 81 stool samples, collected in 28 microscopy-positive and 27 microscopy-negative samples from individuals suspected of intestinal parasitosis and in 26 samples from individuals without suspicion of parasitic infection. The in-house assays detected parasites in more samples from patients suspected of having parasitosis than did any of the kits. We conclude that commercial kits are targeting relevant parasites, but their performance may vary.

## 1. Background

Correct identification of microbial agents causing diarrhoea in humans is crucial for optimal treatment. Detection of disease-causing intestinal parasites is traditionally done by microscopic examination of stool samples. Over the last years this has been changed in favour of using PCR. Studies have shown that both sensitivity and specificity of PCR are better compared to microscopy [1–5]. Moreover, microscopy can lead to false conclusions, with harmless parasites being interpreted as disease-causing, while life-threatening parasites may not be detected. This has in particular been demonstrated for intestinal amoeba [6–10]. For estimating the true impact of parasitic intestinal infections, it is important to establish valid and reliable laboratory techniques for testing stool samples from patients. Use of optimized laboratory methods will improve patient safety through rapid and correct diagnosis, which leads to timely start of appropriate treatment.

The aim of this study was to evaluate the consequences of replacing microscopy by real-time PCR (RT-PCR) for detection of intestinal parasites causing diarrhoea. In order to do so, we first established which parasites were detected by microscopy in our laboratory over a period of nine years, to determine which parasites were relevant in our patient population. We determined which previously detected parasites would be missed by introducing a limited number of species-specific PCR assays and how many cases they represented. We then evaluated the performance of in-house singleplex RT-PCR assays for the three most important intestinal parasitic pathogens. Finally, the performance of three selected in-house RT-PCR assays for detection of* Giardia lamblia*,* Cryptosporidium parvum*/*Cryptosporidium hominis,* and* Entamoeba histolytica* was compared to those of three commercial multiplex real-time PCR (MT-PCR) kits.

Two specific objectives were defined: (1) evaluation of performance of species-specific in-house RT-PCR assays for detection of* G. lamblia*,* C. parvum*/*C. hominis,* and* E. histolytica* in stool samples submitted for examination for parasites; (2) comparison of the performance of the in-house RT-PCR assays with the performance of three commercial MT-PCR kits for detection of the same parasites.

## 2. Methods

### 2.1. Data Collection from the Laboratory Information System (LIS)

Data on faecal samples examined for parasites from October 2005 to January 2015 was extracted from the electronic LIS. The total number of samples and patients and results of microscopy were registered.

### 2.2. Stool Samples

In total 125 stool samples, of which 99 were examined by microscopy on suspicion of parasitosis, were randomly collected from individuals with gastrointestinal complaints between June 2010 and January 2015. Ninety-nine of these samples were included for objective one (31 microscopy-positive and 68 microscopy-negative) and eighty-one (28 microscopy-positive and 27 microscopy-negative) were included for testing objective two. In addition 26 samples from individuals without suspicion of parasitosis were included without microscopy for objective two.

For objective 1, a total of 99 samples were analysed by in-house RT-PCR. For objective 2, a total of 81 samples were analysed by in-house RT-PCR and by three commercial MT-PCR kits. All samples were kept at −80°C until PCR were performed.

### 2.3. Microscopy for Intestinal Parasites

Microscopic examination for the presence of ova and cysts was routinely performed by examination of iodine-stained wet-mount preparations after formalin-ethyl acetate concentration, at a magnification of ×400 [11]. On specific request and when* Cryptosporidium *species,* Cyclospora *species, or* Cystoisospora *species was suspected from routine microscopy a smear stained by modified Ziehl-Neelsen technique was also examined [12].

### 2.4. In-House RT-PCR

For the in-house PCR assays, DNA was extracted by using NucliSENS easyMAG system (bioMérieux, France) in accordance with the manufacturer's instructions. Prior to DNA extraction, a cotton swab was submerged into the stool sample and suspended in 4 ml physiological NaCl solution. An internal extraction and PCR control, phocine herpesvirus (PhHV laboratory strain) was added to each sample prior to DNA extraction [13]. The nucleic acids were eluted in 100 *μ*l and processed for PCR immediately.

Detection of the three intestinal parasites (*G. lamblia, C. parvum*/*C. hominis, *and* E. histolytica*) was performed as singleplex RT-PCR in 99 samples analysed as duplicate. One assay was tested for detection of* G. lamblia*, three were tested for* C. parvum*/*C. hominis*, and one assay (assay 3) was included to distinguish between* C. parvum* and* C. hominis. *Finally one assay for* E. histolytica *was tested, using primers and probes (Table 1) described previously [1, 4, 13–16].

The 25 *μ*l reactions mixture contained 1x TaqMan® Fast Universal PCR Master Mix, 2x No AmpErase® UNG (Thermo Fisher Scientific, Waltham, MA, USA), 1000 nM of the primers, and 200 nM of the probes and 5 *μ*l DNA eluate.

The real-time PCR was performed using an Applied Biosystems 7500 Fast Real-Time PCR Thermocycler (Thermo Fisher Scientific) with the following cycling conditions: 95°C for 20 sec, followed by 45 cycles of 95°C for 3 sec and 60°C for 30 sec.

The PCR products were analysed using Sequence Detection Software v.1.4 (Thermo Fisher Scientific). A manual cycle threshold was set to 0,1 with an automatic baseline. The sample was regarded as positive if the Ct-value was ≤42 and had an exponential curve. Negative and positive extraction and PCR controls were included in all PCR analysis.

The singleplex in-house RT-PCR assays for* G. lamblia*,* C. parvum*/*C. hominis* (assay 1), and* E. histolytica* were collective called kit A and compared to three commercial MT-PCR kits, used in objective 2 on 79 samples.

### 2.5. Diagnostic Test Kits

Three different commercial kits available at the market were tested: RIDA®GENE Parasitic Stool Panel (PG1705) from R-Biopharm AG, Darmstadt, Germany (kit B), LightMix® Modular Gastroenteritis Assays from TIB MOLBIOL, Berlin, Germany (kit C), and BD MAX™ Enteric Parasite Panel from BD Diagnostic, Franklin Lakes, NJ, USA (kit D). DNA extraction for kits B and C was done as described for the in-house assays. For kit D, DNA extraction was done according BD MAX enteric parasite panel instructions on the BD MAX system. In all commercial kits, we used the internal control DNA, which was recommended and included in the kits. Seventy-nine samples were tested in duplicate in all three kits, in accordance with the manufacturer's instructions. For kit B the PCR assays were carried out using an Applied Biosystems 7500 Fast Real-Time PCR Thermocycler with the following cycling conditions: 1 min at 95°C, followed by 45 cycles of 95°C for 15 sec and 60°C for 30 sec. For kit C the PCR assays were done on a Roche LightCycler 480® II real-time instrument with the following cycling conditions: 10 min at 95°C, followed by 50 cycles of 95°C for 5 sec, 62°C for 5 sec, and 72°C for 15 sec. For kit D analysing was done according BD MAX enteric parasite panel instructions on the BD MAX system. A positive result in kits was regarded positive, if one out of two duplicates was positive, used in objective 2.

### 2.6. Analysis

McNemar's test was used for the statistical comparison of the paired data in objective 1.

### 2.7. Ethics, Biobank, and Data Storage

The study is part of a Ph.D. project and approved by the Danish Data Protection Agency (j.nr. 2008-58-0035). All samples were stored at −80°C in an approved research biobank established for the study. Data were stored in an approved and secure portal belonging to Region of Southern Denmark.

## 3. Results

### 3.1. LIS Data

In the period of October 2005 to January 2015, 10593 samples from 4887 patients were examined for intestinal parasites. Based on microscopy the reported diarrhoea-causing parasites were* G. lamblia* (500 samples/237 patients),* Cryptosporidium* species (78 samples/41 patients), and* E. histolytica or E. histolytica/dispar* (159 samples/62 patients) and* Cyclospora *species (25 samples/12 patients). As previously described [10] the majority of parasites reported as* E. histolytica* after microscopy were most probably in fact* E. dispar*, which is considered nonpathogenic. On the basis of these data we decided in the study to test for* G. lamblia* and* C. parvum*/*C. hominis* because of the frequencies by which they cause disease. For* Cryptosporidium* sp. the severe illness it causes in immunocompromised patients and the importance to public health as an agent of food and water borne outbreaks also contributed to the decision. In addition a test for* E. histolytica* was included because amoebic dysentery and amoebic liver abscess are severe conditions requiring immediate and appropriate treatment. Thus only the rare cases of cyclosporiasis (approximately one per year in our diagnostic laboratory) would be missed because of the selection of targets.

### 3.2. Objective 1

The in-house RT-PCR assay for* G. lamblia* was positive in 13 of the 14 microscopy-positive samples and in 5 microscopy-negative samples. All three tested RT-PCR assays for* C. parvum*/*C. hominis* were positive in ten of the eleven microscopy-positive samples as well as in one microscopy-negative sample. The selected species-specific in-house RT-PCR assays detected the expected pathogen in all the microscopy-positive samples except for one sample (number 6, Table 2) which based on microscopy was reported with* G. lamblia *and one sample (number 23, Table 2) with* Cryptosporidium *spp. As none of the RT-PCR assays or MT-PCR kits detected the expected parasites in these samples they may represent false positive microscopy reporting (Table 2). The* Cryptosporidium* assay 3 identified* C. hominis* in four and* C. parvum* in eight samples. The* E. histolytica* RT-PCR assay was only positive in 1 of 8 microscopy-positive samples and none of the microscopy-negative samples. The seven microscopy-positive samples that were negative for* E. histolytica* by PCR were all positive when analysed for* E. dispar* by species-specific PCR.

There was no statistical difference in the number of positive samples when tested by microscopy and PCR for the three selected parasites (*p* = 0,22).

### 3.3. Objective 2

Kit A detected* G. lamblia* in three samples more than kit B, two samples more than kit C, and four samples more than kit D. For* C. parvum*/*C. hominis, *kit A detected one positive sample more than kit B and one positive sample more than kit C but one less than kit D. Only kit D was positive for* C. parvum*/*C. hominis *in sample (number 8) in which the other kits detected* G. lamblia *(Table 2). All kits detected* E. histolytica *in the same sample.

Of all samples tested, discordant results were obtained from duplicate determinations in one sample using in-house assays (kit A), two using kit B, two using kit C, and three using kit D (Table 2).

None of the MT-PCR kits confirmed the presence of* G. lamblia *in sample numbers 32 and 33, which were positive in two out of two and one of two replicates, respectively, by the in-house RT-PCR. These two samples were from the same patient. A sample three days later was positive when tested at the National Reference Laboratory at Statens Serum Institut. Sample numbers 32 and 33 were therefore considered to be true positive but weak.

Sample number 86 (not shown in Table 2) was only tested in kits A, B, and D and therefore not included in the total number. In this sample, kit B was positive for both* G. lamblia *and* E. histolytica,* in one out of two duplicates. This was not confirmed by any of the other test kits.

Inhibition by faecal constituents was not a problem in this study, as it has been reported previously [17].

## 4. Discussion

In this study we have evaluated PCR assays for replacement of microscopy for routine detection of diarrhoea-causing parasites. In contrast to microscopy PCR only detects specific parasites. Careful selection of targets for the PCR assays is therefore mandatory. It is also important to be aware of which diarrhoea-causing parasites present in the population that are not targeted by the selected PCR assays and the number of patients affected by exclusion of assays for particular rare parasites [18, 19].

Based on previous frequencies of detection by microscopy and severity of disease we decided to establish PCR assays for* G. lamblia*,* C. parvum*/*C. hominis,* and* E. histolytica.* The only diarrhoea-causing parasite previously detected and not targeted by the PCR assays was* Cyclospora *spp. with a little more than one case on average each year.

The three different assays for* C. parvum*/*C. hominis* performed equally but assay 1 resulted in the lowest CT values and was used for objective 2. Assay 3 distinguished between* C. hominis* and* C. parvum *and was used for subsequent species identification in positive samples. Rapid species identification is valuable for epidemiological investigations.

The in-house RT-PCR assays detected* G. lamblia *and* Cryptosporidium *spp. in microscopy-negative samples from patients suspected of suffering from intestinal parasitosis and thus appeared more sensitive than microscopy. PCR has previously been reported to be more sensitive than microscopy for detection of specific parasites. In ten Hove's study in 2007, PCR showed 3.6% better sensitivity than microscopy for* Giardia *in clinical stool samples [3], and, in Starks study in 2011, PCR had 2.9% better sensitivity for* Giardia* and 2% better sensitivity for* Cryptosporidium *than microscopy [5]. We found PCR detected 4.1% more* Giardia *than microscopy but did not find any difference for* Cryptosporidium* in this study. A major advantage of PCR over microscopy is the specificity obtained from the discrimination between* E. histolytica* and* E. dispar* [7–10]. Seven of the eight samples originally reported with* E. histolytica* by microscopy were negative in the in-house RT-PCR and were subsequently identified as* E. dispar.*

The comparison of four test kits, including the in-house assay based kit A, showed varying results from replicate tests. Testing in single determinations may lead to false results in a minority of cases. Future use of these assays may be improved by running tests in duplicate.

The limitation of this study was first of all the sample size, which does not allow statistical analysis of differences in performance of microscopy and RT-PCR in-house assays. Tendencies in favour of the in-house assays were seen when comparing variation of replicates and sensitivities to commercial test kits.

As indicated by the numbers of cases of intestinal parasitosis registered in our LIS over the years, collection of larger number of samples will take time. The detection of parasites in microscopy-negative samples suggests that replacement of microscopy with PCR will increase the positive rates and thereby shorten the time needed to establish large sample collections.

## 5. Conclusion

In our setting it is relevant to test for* G. lamblia*,* C. parvum/C. hominis, *and* E. histolytica. *We expect that replacement of microscopy with in-house RT-PCR assays for these parasites will result in higher positive rates for* G. lamblia *and* C. parvum/C. hominis*, while false positive results for* E. histolytica* will be avoided. Addition of a secondary test differentiating between* C. parvum* and* C. hominis* will be of value for early discovery of outbreaks.

All the commercial MT-PCR kits evaluated here tested for the relevant targets. However, some variation in performance was seen when using the kits. The choice of method for detection of intestinal protozoa may depend on the setting. Compared to PCR microscopy is less sensitive and less specific, more time consuming, and more dependent on individual skills. Use of commercial PCR kits may be attractive in laboratories handling moderate numbers of samples, while in-house PCR assays can be established and maintained for large-scale throughput analyses, mainly due to lower costs.

## Figures and Tables

**Table 1:** Primer and probes used for in-house real-time PCR assays in study.

Parasites	Forward primer sequence, 5′→3′	Target	Reference
	Reverse primer sequence, 5′→3′		
	Probe sequence (FAM), 5′→3′		
*Giardia lamblia*	GAC GGC TCA GGA CAA CGG TT TTG CCA GCG GTG TCC G CCC GCG GCG GTC CCT GCT AG	ssu-rRNA	Verweij et al. (2004) [1]

*C. parvum/C. hominis* (assay 1)	CTT TTT ACC AAT CAC AGA ATC ATC AGA TGT GTT TGC CAA TGC ATA TGA A TCG ACT GGT ATC CCT ATA A	DNA J-like protein gene	Bruijnesteijn van Coppenraet et al. (2009) [4]

*C. parvum/C. hominis* (assay 2)	CGC TTC TCT AGC CTT TCA TGA CTT CAC GTG TGT TTG CCA AT CCA ATC ACA GAA TCA TCA GAA TCG ACT GGT ATC	DNA J-like protein gene	Fontaine and Guillot (2002) [14]

*C. parvum/C. hominis* (assay 3)	GAA CTG TAC AGA TGC TTG GGA GAA T CCTT CGT TAG TTG AAT CCT CTT TCC A TTG GAG CTC ATA TCA G *C. hominis* probe CTT GGA GCT CGT ATC AG *C. parvum* probe	Specific protein-coding gene	Yang et al. (2013) [15]

*Entamoeba histolytica*	ATT GTC GTG GCA TCC TAA CTC A GCG GAC GGC TCA TTA TAA CA CAT TGA ATG AAT TGG CCA TT	ssu-rRNA	Verweij et al. (2004) [1] Stensvold et al. (2010) [16]

*Internal control*			

Phocine herpesvirus (PhHV)	GGG CGA ATC ACA GAT TGA ATC GCG GTT CCA AAC GTA CCA A TTT TTA TGT GTC CGC CAC CAT CTG GAT C	Glycoprotein B	Niesters (2001) [13]

**Table 2:** Samples with discordant results obtained from different test kits.

*Sample number*	Mic.	* Giardia*				*Cryptosporidium*			
		Kit A	Kit B	Kit C	Kit D	Kit A	Kit B	Kit C	Kit D
6	Giardia	0/0	0/0	0/0	0/0	0/0	0/0	0/0	0/0
7	Giardia	+/+	0/0	**+/0**	+/+	0/0	0/0	0/0	0/0
8	Giardia	+/+	+/+	+/+	0/0	0/0	0/0	0/0	**+/0**
9	Giardia	+/+	+/+	+/+	**+/0**	0/0	0/0	0/0	0/0
12	Giardia	+/+	+/0	**+/0**	0/0	0/0	0/0	0/0	0/0
15	Crypto	0/0	0/0	0/0	0/0	+/+	0/0	+/+	+/+
18	Crypto	0/0	0/0	0/0	0/0	+/+	+/+	**+/0**	+/+
23	Crypto	0/0	0/0	0/0	0/0	0/0	0/0	0/0	0/0
24	Crypto	0/0	0/0	0/0	0/0	+/+	+/+	0/0	+/+
32	undet	+/+	0/0	0/0	0/0	0/0	0/0	0/0	0/0
33	undet	**+/0**	0/0	0/0	0/0	0/0	0/0	0/0	0/0
34	undet	+/+	+/+	+/+	+/+	0/0	0/0	0/0	0/0
35	undet	+/+	+/+	+/+	+/+	0/0	0/0	0/0	0/0
42	undet	0/0	0/0	0/0	0/0	+/+	+/+	+/+	+/+
49	undet	+/+	+/+	+/+	**+/0**	0/0	0/0	0/0	0/0

*Total positive*		**9**	**6**	**7**	**5**	**4**	**3**	**3**	**5**

Mic = microscopy; undet = undetected; + = positive; 0 = negative. Results from duplicate tests are shown as +/+, **+**/**0**, and 0/0. Discordant results are in bold. Kit A = in-house RT-PCR assays, kit B = RIDA GENE Parasitic Stool Panel (PG1705), kit C = LightMix Modular Gastroenteritis Assays, and kit D = BD MAX Enteric Parasite Panel.
